# Estimating the under-reporting of norovirus illness in Germany utilizing enhanced awareness of diarrhoea during a large outbreak of Shiga toxin-producing *E. coli* O104:H4 in 2011 – a time series analysis

**DOI:** 10.1186/1471-2334-14-116

**Published:** 2014-03-01

**Authors:** Helen Bernard, Dirk Werber, Michael Höhle

**Affiliations:** 1 Department for Infectious Disease Epidemiology, Robert Koch Institute, Seestraße 10, 13353 Berlin, Germany; 2 Department of Mathematics, Stockholm University, 106 91 Stockholm Sweden

**Keywords:** Norovirus, Gastroenteritis, Epidemiology, Disease notification, Time series analysis, Public health, Population surveillance, Under-reporting

## Abstract

**Background:**

Laboratory-confirmed norovirus illness is reportable in Germany since 2001. Reported case numbers are known to be undercounts, and a valid estimate of the actual incidence in Germany does not exist. An increase of reported norovirus illness was observed simultaneously to a large outbreak of Shiga toxin-producing *E. coli* O104:H4 in Germany in 2011 – likely due to enhanced (but not complete) awareness of diarrhoea at that time. We aimed at estimating age- and sex-specific factors of that excess, which should be interpretable as (minimal) under-reporting factors of norovirus illness in Germany.

**Methods:**

We used national reporting data on laboratory-confirmed norovirus illness in Germany from calendar week 31 in 2003 through calendar week 30 in 2012. A negative binomial time series regression model was used to describe the weekly counts in 8∙2 age-sex strata while adjusting for secular trend and seasonality. Overall as well as age- and sex-specific factors for the excess were estimated by including additional terms (either an O104:H4 outbreak period indicator or a triple interaction term between outbreak period, age and sex) in the model.

**Results:**

We estimated the overall under-reporting factor to be 1.76 (95% CI 1.28-2.41) for the first three weeks of the outbreak before the outbreak vehicle was publicly communicated. Highest under-reporting factors were here estimated for 20–29 year-old males (2.88, 95% CI 2.01-4.11) and females (2.67, 95% CI 1.87-3.79). Under-reporting was substantially lower in persons aged <10 years and 70 years or older.

**Conclusions:**

These are the first estimates of (minimal) under-reporting factors for norovirus illness in Germany. They provide a starting point for a more detailed investigation of the relationship between actual incidence and reporting incidence of norovirus illness in Germany.

## Background

Noroviruses are the most frequent cause of acute gastroenteritis and foodborne illness [1-3]. Norovirus illness is usually self-limiting and of short duration. However, severe courses of disease occur among vulnerable sub-populations and large case numbers have a high impact on the population level [4,5].

In Germany, laboratory-confirmed norovirus infection is reportable according to the Protection Against Infection Act of 2001. Laboratories report the detection of norovirus to the local public health departments, which further investigate these cases. Anonymised case-based data are forwarded by the local public health department in an electronic format via one of the 16 state health authorities to the Robert Koch Institute (RKI) at the national level [6] where it is available in an electronic database. The annual reported incidence of norovirus illness was 142 cases per 100,000 population in 2011 [7]. Reported case numbers are, however, known to be undercounts because only a proportion of symptomatic persons consults physicians, and laboratory testing for norovirus is furthermore only initiated on a proportion of these [8]. No study thus far has attempted to estimate under-reporting of norovirus illness in Germany.

In May-July 2011, Germany faced a large outbreak of Shiga toxin-producing *E. coli* (STEC) O104:H4 infections, causing an unprecedented number of cases of hemolytic-uremic syndrome [9]. The outbreak centred in Northern Germany, but affected the entire country; media coverage was also nationwide. Diarrhoea was one of the earliest disease symptoms in cases. Early during the outbreak the public was informed that a large proportion of cases occurred in previously healthy women and was advised to consult a physician when developing bloody diarrhoea.

**Figure 1:**
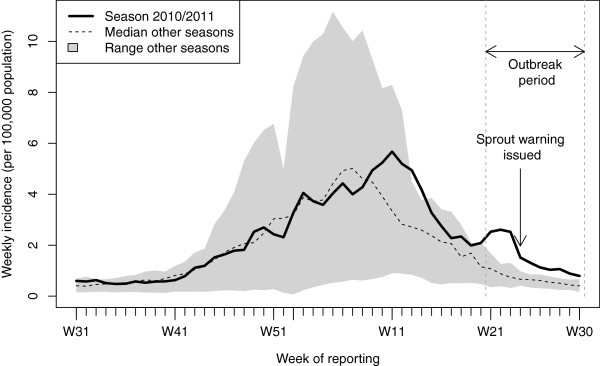
Reporting incidence of laboratory-confirmed norovirus illness in season 2010/2011 (continuous line) and median reporting incidence (dashed line) and range (minimum to maximum, grey area) of the other eight seasons (2003/2004 through 2009/2010 and 2011/2012) by week of reporting, Germany.

In retrospect, an increase of reported case numbers was observed for norovirus and other gastrointestinal pathogens during the outbreak period compared to the same time period in preceding and subsequent years (Figure 1). We hypothesized that this excess was due to enhanced awareness of diarrhoea leading to a more complete ascertainment of norovirus illnesses in the German reporting system. The objective of this study was to estimate the magnitude of the reporting excess during the time-period of heightened media attention of the STEC O104:H4 outbreak, i.e. in weeks 21–30 covering late May, June and July, overall and specific for sex and age-groups. We were particularly interested in the first three weeks of the STEC outbreak, i.e. weeks 21–23, when there was still uncertainty about the outbreak vehicle, assuming that the reporting excess would be highest during this time period.

## Methods

We extracted data on all laboratory-confirmed cases of norovirus illness reported during nine norovirus seasons from week 31, 2003, through week 30 in 2012 from the national surveillance database (date of query: 27 August 2012). The data is freely available via http://www3.rki.de/SurvStat/. Since norovirus activity peaks during wintertime, a season was considered to range from week 31 in one year through week 30 in the following year.

Case numbers were aggregated by year and week of reporting, eight age-groups and sex, resulting in 9∙52∙8∙2 = 7488 observations to be included in the analyses. Cases reported in weeks 53 in the years 2004 and 2009, were randomly distributed to week 52 of the same or week 1 of the following year, respectively.

We conducted a count data time series analysis for the weekly case number using a negative binomial regression model [10] containing population offsets, sine-cosine terms and age and sex as stratification variables (see Additional file 1: Mathematical Appendix for details). The reporting excess during the outbreak was parameterised in the model as a binary indicator for O104:H4 outbreak week (i.e., weeks 21 to 30 in 2011). The rationale for choosing these weeks was that the public was informed about the outbreak in week 21 of 2011 [11] and the outbreak was publicly declared over in week 30 of 2011 [12]. In additional analyses, we divided this ten-week period into a 3-week period before the public communication that sprouts were the likely outbreak vehicle (at the end of week 23 of 2011) [13] and the subsequent 7-week period. To additionally obtain age- and sex-specific estimates of the excess, we also fitted a model containing a triple interaction term (outbreak period, age and sex).

All analyses were performed with Stata 12 (StataCorp. College Station, TX) and R version 3.0.2 (R Foundation for Statistical Computing. Vienna, Austria). The study did not require ethical approval.

## Results

A total of 748,753 laboratory-confirmed cases of norovirus illness were included in the analysis. During the ten-week period of increased media attention due to the STEC outbreak (weeks 21 through 30, 2011), a total of 12,588 laboratory-confirmed norovirus cases were reported in season 2010/2011 compared to a median of 5,584 (range 2,915-7,798) in the non-outbreak seasons. This corresponds to a weekly incidence of 1.54 cases/100,000 population in season 2010/2011 compared to an average weekly incidence of 0.68 cases/100,000 population in the non-outbreak seasons. The age- and sex-stratified under-reporting factors derived from the weekly incidences ranged from 1.77 to 3.48 (Table 1).

**Table 1:** **Age- and sex-stratified frequency and weekly incidence of norovirus case reports**^**§ **^**during calendar weeks 21–30 - Comparison of season 2010/2011 with seasons 2003/2004 through 2009/2010 and 2011/2012, Germany**

	**Both sexes**					**Males**					**Females**				
	**Cumulative case number**		**Average weekly incidence W21-W30 [cases/100,000 population]**			**Cumulative case number**		**Average weekly incidence W21-W30 [cases/100,000 population]**			**Cumulative case number**		**Average weekly incidence W21-W30 [cases/100,000 population]**		
	**W21-W30**					**W21-W30**					**W21-W30**				
**Age [years]**	O104– outbreak season* [n]	Non-outbreak seasons** [Median (range)]	O104– outbreak season* [n]	Non-outbreak seasons** [Median (range)]	**Under-reporting factor**	O104– outbreak season* [n]	Non- outbreak seasons** [Median (range)]	O104– outbreak season* [n]	Non-outbreak seasons** [Median (range)]	**Under-reporting factor**	O104– outbreak season* [n]	Non-outbreak seasons** [Median (range)]	O104– outbreak season* [n]	Non-outbreak seasons** [Median (range)]	**Under-reporting factor**
0-9	2,695	1,493 (861–1,955)	3.86	2.12 (1.16-2.73)	1.82	1,441	820 (475–1,035)	4.03	2.28 (1.25-2.82)	1.77	1,254	660.5 (386–920)	3.69	1.93 (1.07-2.64)	1.92
10-19	841	321.5 (154–462)	1.04	0.38 (0.18-0.57)	2.76	419	161.5 (75–252)	1.01	0.36 (0.17-0.61)	2.78	422	157 (79–214)	1.07	0.37 (0.18-0.54)	2.93
20-29	1,380	407 (209–637)	1.39	0.41 (0.21-0.64)	3.37	604	178 (92–273)	1.19	0.36 (0.19-0.54)	3.36	776	222.5 (117–365)	1.59	0.46 (0.24-0.75)	3.48
30-39	1,046	357 (209–471)	1.07	0.35 (0.19-0.47)	3.03	467	164.5 (71–217)	0.94	0.31 (0.12-0.43)	3.00	579	189 (138–255)	1.20	0.38 (0.24-0.53)	3.16
40-49	1,187	453.5 (236–674)	0.86	0.33 (0.18-0.49)	2.65	528	211 (87–319)	0.75	0.30 (0.13-0.46)	2.54	659	240.5 (149–355)	0.98	0.35 (0.22-0.53)	2.77
50-59	1,248	419 (200–738)	1.07	0.37 (0.19-0.63)	2.88	564	186.5 (83–359)	0.96	0.33 (0.17-0.61)	2.90	684	225.5 (109–379)	1.17	0.40 (0.20-0.65)	2.95
60-69	1,038	460 (227–701)	1.15	0.50 (0.23-0.78)	2.31	493	219 (96–336)	1.12	0.49 (0.19-0.77)	2.31	545	234.5 (124–365)	1.17	0.50 (0.25-0.79)	2.35
70+	3,153	1,504 (564–2,498)	2.53	1.28 (0.55-2.00)	1.98	1,252	527 (172–957)	2.46	1.12 (0.45-1.88)	2.20	1,901	977 (392–1,541)	2.58	1.38 (0.61-2.09)	1.86
All	12,588	5,584 (2,915-7,798)	1.54	0.68 (0.35-0.95)	2.26	5,768	2,567 (1,277-3,557)	1.44	0.64 (0.32-0.89)	2.25	6,820	3,017 (1,638-4,241)	1.64	0.72 (0.39-1.02)	2.27

Note. *W* calendar week; *CI* confidence interval; * season 2010/2011, in which a large outbreak of Shiga toxin-producing *E. coli* O104:H4 occurred; ** seasons 2003/2004 through 2009/2010 and 2011/2012.

^§^Laboratory-confirmed cases.

Using multivariable regression modelling for the entire time series, we estimated the multiplication factor for the overall under-reporting to be 1.51 (95% confidence interval (CI) 1.17-1.96) for the ten-week period. For the three-week period before and the seven-week period after the public communication of sprouts as the likely outbreak vehicle the factors were 1.76 (95% CI 1.28-2.41) and 1.39 (95% CI 1.05-1.83), respectively. In stratified analyses, the highest factors for the ten-week period were 2.63 (95% CI 2.11-3.27) and 2.28 (95% CI 1.83-2.82) in 20–29 year-old males and females, respectively (Table 2). Factors for the three-week period were even higher with 2.88 (95% CI 2.01-4.11) and 2.67 (95% CI 1.87-3.79) in 20–29 year-old males and females, respectively. For the youngest and the oldest age-groups, i.e. 0–9 year-olds and those aged 70 years and above, the under-reporting factors were consistently low and did not differ significantly from 1. This holds true for males and females and was seen for the entire ten-week period as well as the three-week and the seven-week period, the only exception being the three-week period under-reporting factor for males aged 70 years and above.

**Table 2:** **Estimated under-reporting factors for norovirus illness by age-group and sex during a large outbreak of Shiga toxin-producing *E. coli *** **O104, Germany, 2011**

	**W21-W30**						**W21-W23**						**W24-W30**					
	**Males**			**Females**			**Males**			**Females**			**Males**			**Females**		
**Age [years]**	**Factor**	**95% CI**		**Factor**	**95% CI**		**Factor**	**95% CI**		**Factor**	**95% CI**		**Factor**	**95% CI**		**Factor**	**95% CI**	
0-9	1.16	0.94	1.43	1.18	0.95	1.45	1.22	0.86	1.72	1.34	0.94	1.89	1.10	0.86	1.40	1.07	0.83	1.37
10-19	2.08	1.66	2.62	1.96	1.56	2.46	2.49	1.73	3.58	2.18	1.51	3.13	1.83	1.39	2.40	1.80	1.37	2.35
20-29	2.63	2.11	3.27	2.28	1.83	2.82	2.88	2.01	4.11	2.67	1.87	3.79	2.43	1.87	3.15	2.03	1.57	2.62
30-39	2.25	1.80	2.81	2.09	1.68	2.61	2.52	1.75	3.61	2.55	1.78	3.64	2.05	1.57	2.68	1.82	1.40	2.37
40-49	1.88	1.50	2.34	1.79	1.44	2.23	2.42	1.69	3.46	2.02	1.41	2.88	1.57	1.20	2.05	1.63	1.26	2.11
50-59	1.74	1.40	2.17	1.75	1.41	2.18	2.00	1.40	2.86	2.03	1.43	2.90	1.57	1.20	2.04	1.57	1.21	2.04
60-69	1.30	1.04	1.63	1.47	1.18	1.84	1.61	1.12	2.30	1.72	1.20	2.46	1.12	0.85	1.46	1.31	1.00	1.70
70+	1.12	0.91	1.39	0.96	0.78	1.18	1.44	1.02	2.03	1.19	0.84	1.67	0.95	0.74	1.22	0.83	0.65	1.06

Note. *W* calendar week; *CI* confidence interval.

## Discussion

We estimated the magnitude of the reporting excess of laboratory-confirmed norovirus illness in Germany that paralleled a large outbreak of STEC O104:H4. Based on a count data time series analysis, the weekly incidence of reported norovirus illness increased overall by a factor of 1.76 (i.e., 76%) for the first three weeks of the outbreak before the outbreak vehicle was publicly communicated, with the highest excess in males aged 20–29 years (factor 2.88). Because it is highly unlikely that all persons suffering from gastroenteritis consulted a physician and received laboratory testing for norovirus during this outbreak, we interpret these estimates to be minimal under-reporting factors. This explains – apart from differing structures of surveillance systems – why our estimated overall under-reporting factor is lower than factors reported from other countries, e.g. factor 12.7 from England and Wales [8].

The estimated under-reporting varied by sex and by age-group and was highest in 20–29 year-olds. However, it was not as high as we had expected based on the fact that younger age-groups, especially women, were communicated to be highly represented among cases early during the outbreak. Our assumption was that especially women aged 20–39 years presented more frequently to their physician when suffering from gastroenteritis during the outbreak than usually. There are two possible explanations: either, the public, and especially the younger age-groups, did not perceive the risk communication message as it was intended, and patients did not consult physicians more often when suffering from diarrhoea, or physicians did not initiate laboratory testing more often for these younger age-groups. Alternatively, the message was perceived and physician consultations and laboratory testing increased, but norovirus infections were not that prevalent in this age-group. For the youngest (0–9 years) and the oldest age-group (70 years and above), the differences were less pronounced, or even absent, compared to previous years. The discrepancy between the crude reporting excess reported in Table 1 and the (smaller) model-based under-reporting factors for these two age-groups can be explained by the increasing trend of reported case numbers over time (see Additional file 1: Mathematical Appendix). Furthermore, we did not find any significant geographic variation of our estimates (data not shown). The Mathematical Appendix also contains a more detailed analysis and visualisation of the model fit together with a discussion of possible model limitations including autocorrelation.

Our approach is unusual in that it used data from the passive surveillance system of infectious diseases to estimate this systems’ own degree of under-ascertainment (with regard to norovirus illness). Enabled by the occurrence of a public health emergency event, this inexpensive approach cannot distinguish between the effects of increased health care-seeking behaviours by cases, stool collection by clinicians, or testing by laboratories. It furthermore remains unclear whether physicians who collected patients’ stool during the outbreak specifically ordered testing for norovirus or whether they simply ordered testing for a panel of infectious enteric pathogens, which then also included norovirus. The observed parallel reporting increase of other enteric pathogens, e.g., Campylobacter spp., gives weight to the hypothesis that norovirus testing was often an unintentional by-product of increased stool testing initiated to search primarily for STEC during the outbreak. How detailed physicians need to specify the pathogens when ordering laboratory testing is influenced by health insurance reimbursement policies, which vary across Germany. At any rate, more complex study designs are needed, e.g., cohort studies, to specifically address under-reporting at the different levels of the surveillance pyramid.

We deem it unlikely that the increase in reported case numbers reflects a true increase in norovirus activity in the outbreak period. First, increased case numbers were also reported for other reportable infectious enteric diseases for this period [7], supporting our hypothesis that fear of STEC infection led to more diagnostic testing. Second, the increase was higher before the public announcement that sprouts were the likely vehicle of infection, suggesting less pressure for testing after the announcement. Third, the increase differed across age-groups. It was less pronounced in young children and older women who usually have the highest incidences of reported norovirus illness [14] due to their exposure in child-care facilities and residential homes, whereas it was stronger in young adults who are usually not that frequently affected in these classical norovirus outbreak settings. A fourth argument against a true increase in norovirus activity during the outbreak period is that, although large fluctuations of the incidence of norovirus illness between the seasons exist and have been hypothesized to be influenced by the emergence of new virus variants [15], the season 2010/2011 altogether was one with lower norovirus activity compared to 2009/2010 in Germany [7] and other countries in Europe, e.g. in England and Wales [16].

Due to the temporal occurrence of the STEC O104:H4 outbreak, our estimates apply to a specific time-period outside the peak of the norovirus season (which is classically in winter). Hence, our analyses cannot show, whether under-reporting factors are time dependent and, if so, in what direction they would tend to go in other time periods, e.g. during the peak season. It is conceivable that they were smaller during winter because the health-care system would be more aware of norovirus infections and more likely to detect them. On the other hand, increased norovirus circulation and a higher familiarity with gastroenteritis symptoms during winter could lead to lower consultation rates of patients and stool collection rates by physicians, and therefore to larger under-reporting.

## Conclusions

In summary, we estimated minimal age- and sex-specific under-reporting factors for norovirus illness in Germany outside the peak season. The magnitude of under-reporting varied by sex and age-group; it was highest in 20–29 year-olds (factor 2–3), a targeted age-group of public advices during the STEC O104:H4 outbreak, and was basically not observed in persons aged <10 years and 70 years or older. Our results provide a starting point for investigating in more detail the relationship between the actual incidence and the reporting incidence of norovirus illness in Germany.

## Competing interests

The authors declare that they have no competing interests.

## Authors’ contributions

HB conceived of the study, participated in the study design, the statistical analyses, the data interpretation and the manuscript drafting. DW participated in the data interpretation and the manuscript drafting. MH participated in the study design, the statistical analyses, the data interpretation and the manuscript drafting. All authors read and approved the final manuscript version.

## Pre-publication history

The pre-publication history for this paper can be accessed here:

http://www.biomedcentral.com/1471-2334/14/116/prepub

## Supplementary Material

Additional file 1 The Mathematical Appendix contains details on the statistical modelling including a more detailed analysis and visualisation of the model fit together with a discussion of possible model limitations. Click here for file
