# Supplementary material for: Estimating the under-reporting of norovirus illness in Germany utilizing enhanced awareness of diarrhoea during a large outbreak of Shiga toxin-producing E. coli O104:H4 in 2011 – a time series analysis
Source: BMC Infect Dis. 2014 Mar 1;14:116. doi: 10.1186/1471-2334-14-116 (PMC3941256; doi:10.1186/1471-2334-14-116)
Supplement: Additional file 1 — The Mathematical Appendix contains details on the statistical modelling including a more detailed analysis and visualisation of the model fit together with a discussion of possible model limitations. [file 1471-2334-14-116-S1.pdf]

Supplementary Data
Mathematical Appendix for the Statistical Modelling

Helen Bernard, Dirk Werber and Michael Höhle

February 19, 2014

Abstract

This document contains the details of the negative binomial model used to determine under-reporting factors in the paper *Estimating the under-reporting of norovirus illness in Germany utilizing enhanced awareness of diarrhoea during a large outbreak of Shiga toxin-producing E. coli O104:H4 in 2011 - a time series analysis* published in BMC Infectious Diseases.

1 Introduction

Aim of our statistical modelling was to provide a model based baseline for the number of cases, which we would have expected, had there been no STEC O104:H4 outbreaks during the weeks W21-W30 in 2011. Of course, this could be done by simple methods such as removing the W21-W30 data in 2011, then fit any type of curve (line, polynomial, spline) to the remaining data of 2011 and finally take this extrapolation as baseline for W21-W30 in 2011. However, we want to borrow information on the shape from previous and future norovirus seasons. Hence, time series modelling is a good way to determine not only this baseline, but also directly represent the under-reporting factor as part of the model. Furthermore does this model based approach allow a direct calculation of confidence intervals for the factors.

2 Statistical Modelling

We investigated the age- and sex-stratified count data time series using a negative binomial regression model with a stratum-specific mean μ and variance $\mu + d\mu^2$, where $d > 0$ is a single dispersion parameter common for all strata. The probability mass function under this parametrization of the negative binomial distribution can be found, for example, as equation (2.1) in [1]. The reported number of cases of norovirus illness in week $t = 1, \ldots, 468$, age-group a (0-9, 10-19, 20-29, 30-39, 40-49, 50-59, 60-69, ≥70 years) and sex s (male/female) is now given by

$$y_{tas} \sim \text{NegBin}(\mu_{tas}, d),$$

where

$$\log(\mu_{tas}) = \log(n_{tas}) + g(t) + \beta_{\text{season}(t)} + \gamma_a + \delta_s + \eta_{as} \cdot \text{O104}(t), \tag{1}$$

and

$$\begin{aligned} g(t) = {} & \alpha_{1,\text{season}(t)} \sin\left(\frac{2\pi t}{52}\right) + \alpha_{2,\text{season}(t)} \cos\left(\frac{2\pi t}{52}\right) \\ & + \alpha_{3,\text{season}(t)} \sin\left(\frac{4\pi t}{52}\right) + \alpha_{4,\text{season}(t)} \cos\left(\frac{4\pi t}{52}\right). \end{aligned} \tag{2}$$

Here, season(t) is a function giving the norovirus season (i.e. it returns a number between 1 and 9 representing the nine seasons 2003/2004–2011/2012) that week t belongs to. Furthermore, O104(t) is a binary indicator function which is one if week t is in the time interval from 2011-W21 to 2011-W30 (i.e. the Shiga toxin-producing E. coli O104:H4 outbreak period) and zero otherwise. Finally, n_{tas} is the German population size in strata (t, a, s), where we have used a centered five-week moving average on the yearly data available from [2]. Thus, the nine coefficients $\beta_{2003/2004} - \beta_{2011/2012}$ describe the magnitude of each season, the eight γ's denote the main effect of each age-group and the δ_s denote the main effect of female sex. Note that in the actual model one of the categories of the variables season, age-group and sex was used as base-line, i.e. a dummy encoding of the factor variables was used.

Altogether, the multiplicative increase in the expected number of reported cases in age-group a and sex s during the outbreak weeks is given as

$$\exp(\eta_{as}).$$

We refer to this estimate as the (model based) *multiplication factor* for under-reporting. It is to be interpreted as a lower bound for the compound under-reporting across all levels of the reporting pyramid. Note: Since we use $\log(n_{tas})$ as offset in (1), $\exp(\eta_{as})$ is also the multiplicative increase in the expected reporting incidence in age-group a and sex s during the outbreak weeks. The overall factor for under-reporting without age-sex stratification is computed in the model by assuming that there is one common η for the age-sex groups, i.e. $\forall a, s : \eta_{as} = \eta$.

Finally, $(1 - \alpha) \cdot 100\%$ Wald confidence intervals (CIs) for $\exp(\eta_{as})$ or $\exp(\eta)$ are obtained by exp-transforming the limits of the respective $(1 - \alpha) \cdot 100\%$ Wald CIs for η_{as} and η. Note that these CIs only reflect the uncertainty obtained by fitting the selected time series model to the observed time series data including the period of enhanced awareness of diarrhoea during a large outbreak of Shiga toxin-producing E. coli O104:H4 in 2011. Hence as explained in the manuscript the estimated under-reporting factors are only lower bounds for the (unknown) under-reporting. Hence, the same applies for the CIs of our estimates.

2.1 Dividing the STEC O104:H4 outbreak period into two periods

In additional analyses we divided the ten-week outbreak period into a three-week period (2011-W21 to 2011-W23) *before* and a seven-week period (2011-W24 to 2011-W30) *after* the public communication of sprouts as the likely outbreak vehicle at the end of week 2011-W23 [3]. In other words, $\eta_{as} \cdot \text{O104}(t)$ in (1) is replaced by

$$\eta_{as}^{\text{before}} \cdot \text{O104}_{\text{before}}(t) + \eta_{as}^{\text{after}} \cdot \text{O104}_{\text{after}}(t),$$

and our hypothesis is that $\eta_{as}^{\text{before}} > \eta_{as}^{\text{after}}$.

3 Model fit

The $g(t)$ described in (2) represents an interaction between a function consisting of two harmonics and a factor representing the season. This representation was obtained by a model selection procedure based on the Bayesian Information Criterion (BIC): For $g(t)$ we compared models with 1-3 harmonics and with or without an interaction with the season factor. Furthermore, we also tried a cyclic P-spline as part of a generalized additive negative binomial model as fitted by the R package `mgcv` [4] (details omitted).

Figure 1 illustrates the model fitted aggregated weekly incidence from the model with and without interaction term between harmonics and season factor (termed `m.nb.2h` and `m.nb.2hXs`, respectively). The model fit shown in the figure are for the model containing one factor per age-sex group for the entire W21-W30 outbreak period in 2011, i.e. corresponding to the model of the first part of Table 2 in the main manuscript. The dotted lines for W21-W30 in 2011 show the model computed baseline. For comparison, also the weekly number of reported cases is shown.

Figure 2 and Figure 3 show the 2×8 age-gender time series for the 2010/2011 season together with the median per week of the non-2010/2011 seasons. Finally, the expected number of cases as given by the `m.nb.2hXs` model is shown.

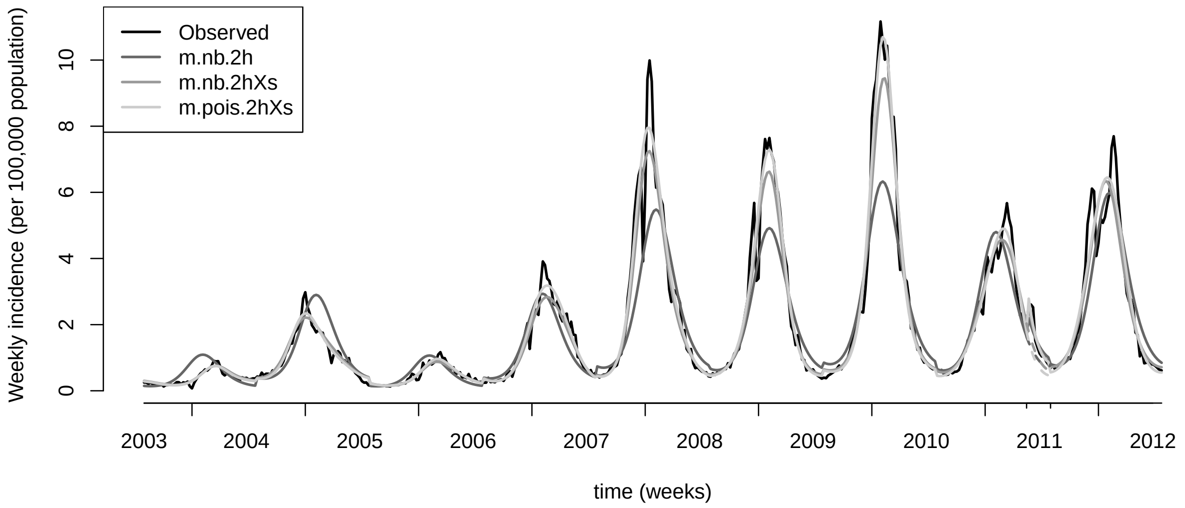

Figure 1: Observed weekly incidence aggregated over all age-sex groups and the expectation of the fitted model(s). The two short axis ticks in 2011 indicate the start and end of the STEC O104:H4 outbreak period. Also shown is the expected incidence of three competing models to be explained in the text.

In the two figures we observe, that the model for age-group 0-9 years for both males and females appears to overshoot a little, while the fit for the other age-groups look fine. One possible explanation for the overshooting is that the increase and decrease of incidence in this age-group appears to be more rapid and the peak period longer than captured by the harmonics. However, the figures also makes clear that our modelling provides a more adequate baseline than the median from the non-2010/2011 seasons, which results in a too low baseline. Hence, the raw under-reporting factors computed in Table 1 of the main paper are too high, because the 2010/2011 season apparently began later than usual and also had more activity than the median of the other seasons (especially, because seasons 2003/2004–2006/2007 had lower activity).

4 Model discussion

We chose a negative binomial regression model in order to take the count data nature of the available surveillance time series into account. This is especially favourable in scenarios with few counts per week in some strata, since the negative binomial distribution here reflects the true count data nature of the data better than modelling, e.g., the log-incidence in a Gaussian framework. While the selected negative binomial model described by equations (1)-(2) had a *much* better overall BIC than the same model using the Poisson as response distribution, the estimated age-sex specific under-reporting factors were between 12-66% higher for the Poisson model (10 week outbreak period). However, this is partly because the expectation of the Poisson is higher in the season peaks and lower in the off-season periods, as this is the only way for it to fit the data (see Fig. 1). Thus, some of the reporting excess during the STEC O104:H4 outbreak is in the negative binomial just explained as additional variation and put into the dispersion parameter. However, since we – as explained below – do not take the possible auto-correlation of the residuals additionally into account the extra-variation of the negative binomial is a good implicit way to address this. Altogether, we favour the negative binomial due to its much better BIC value.

By employing a time series approach we could take into account that the norovirus activity during each season is of varying magnitude. Had we performed the analysis using only the W21-W30 counts of each year, we would not have been able to distinguish whether the increased activity during 2011 was due to a large

season or due to an excess of reporting.

A trend component including season-specific activity levels and harmonic components was used in order to address the time series nature of the data. Remaining auto-correlation of the residuals, as possibly hinted by Figures 1–3, was not additionally addressed. A consequence is thus that the independence assumption of the residuals (i.e. response variables are independent given the model) is slightly violated and hence the reported confidence intervals for the multiplication factors might be too narrow. However, addressing auto-correlation in multivariate count-data time series analysis is not straightforward. See, e.g., [5] for an auto-regressive order 1 negative-binomial time series approach to address this problem. Another approach would be to use a vector ARMA model for the age-sex stratified log-incidences, but since such a model operates on an assumption of Gaussian errors, the count data nature would not be taken into account. Since the aim of our time series modelling was to use the other seasons for estimating the baseline for the 2010/2011 season, we think ignoring the additional auto-correlation is acceptable. Thus for clarity in presentation and communication we have refrained from performing such an extended analysis.

5 References

1. Lawless, J.F.: Negative Binomial and Mixed Poisson Regression, The Canadian Journal of Statistics, 1987, 15(3):209-225.

2. German population by federal states, age and sex [in German] [https://www-genesis.destatis.de/genesis/online]

3. New findings on the STEC outbreak [in German] [http://www.rki.de/DE/Content/Service/Presse/Pressemitteilungen/2011/08_2011.html]

4. Wood, S N: Generalized Additive Models: An Introduction with R. Chapman and Hall/CRC, 2006.

5. Held L, Höhle M, Hofmann M: A statistical framework for the analysis of multivariate infectious disease surveillance data. Statistical Modelling, 2005, 5:187-199.

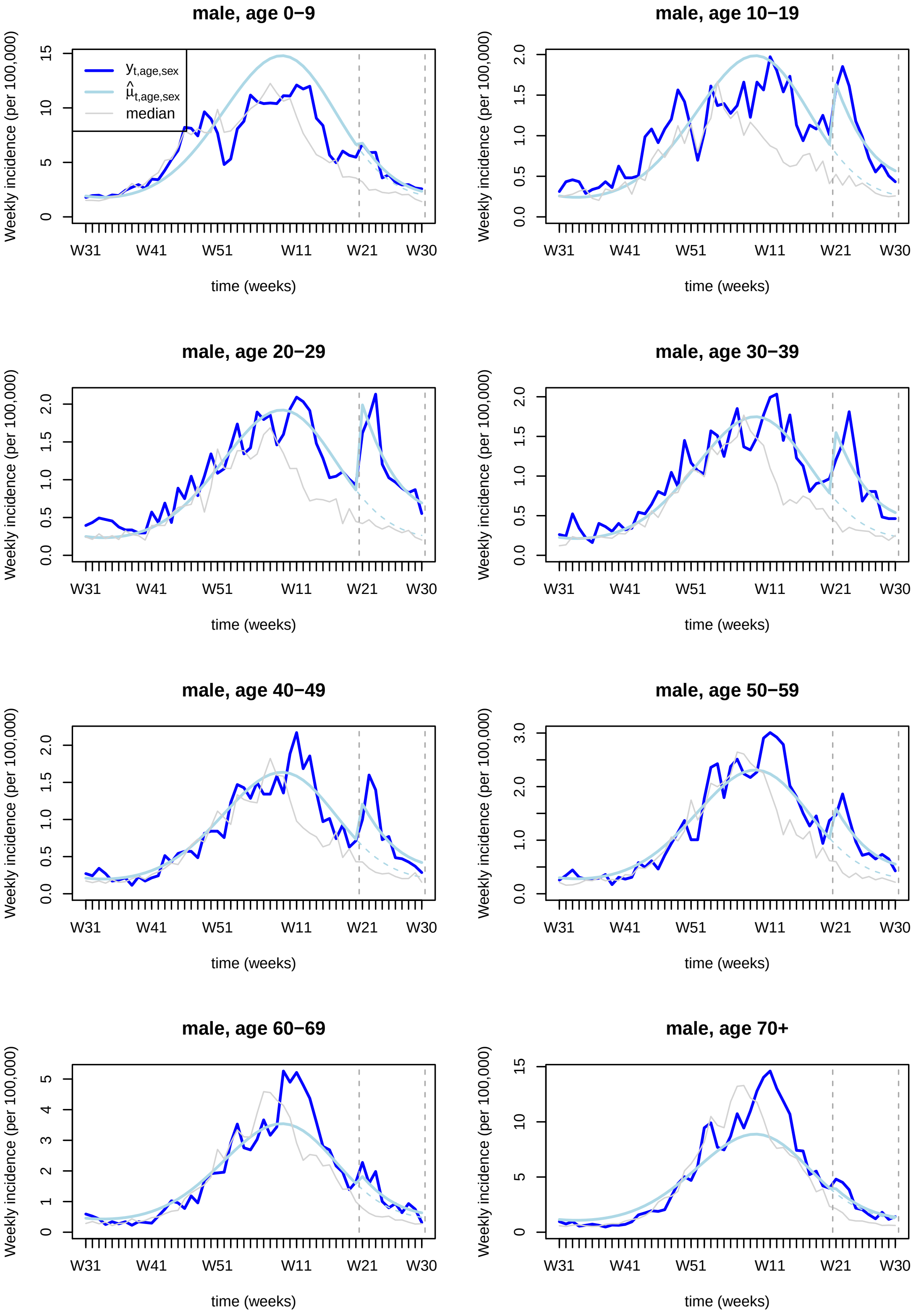

Figure 2: Observed weekly incidence among males during the season 2010/2011 in each of the 8 age strata. Also shown is the expected number of cases from the fitted model. Note the different scaling of the y-axes.

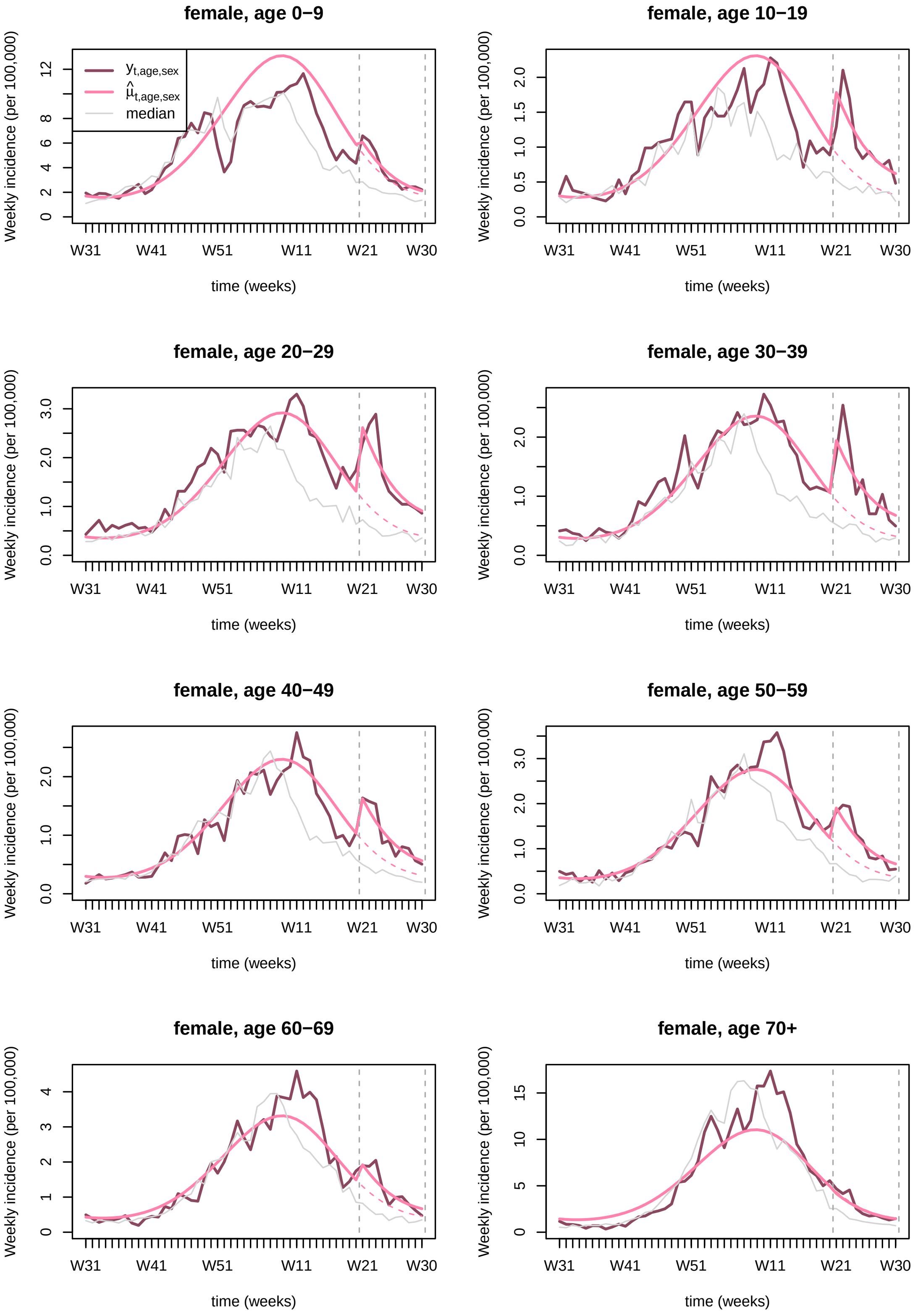

Figure 3: Observed weekly incidence among females during the season 2010/2011 in each of the 8 age strata. Also shown is the expected number of cases from the fitted model. Note the different scaling of the y-axes.
